# The effectiveness of intervention with board games: a systematic review

**DOI:** 10.1186/s13030-019-0164-1

**Published:** 2019-10-21

**Authors:** Shota Noda, Kentaro Shirotsuki, Mutsuhiro Nakao

**Affiliations:** 1 Graduate School of Human and Social Sciences, Musashino University, 3-3-3 Ariake, Koutouku, Tokyo, 135-8181 Japan; 2 Faculty of Human Sciences, Musashino University, Tokyo, Japan; 3 Department of Psychosomatic Medicine, School of Medicine, International University of Health and Welfare, Chiba, Japan

**Keywords:** Board game, Systematic review, Education, Cognitive function

## Abstract

To examine the effectiveness of board games and programs that use board games, the present study conducted a systematic review using the PsycINFO and PubMed databases with the keywords “board game” AND “trial;” in total, 71 studies were identified. Of these 71 studies, 27 satisfied the inclusion criteria in terms of program content, intervention style, and pre–post comparisons and were subsequently reviewed. These 27 studies were divided into the following three categories regarding the effects of board games and programs that use board games: educational knowledge (11 articles), cognitive functions (11 articles), and other conditions (five articles). The effect sizes between pre- and post-tests or pre-tests and follow-up tests were 0.12–1.81 for educational knowledge, 0.04–2.60 and − 1.14 – − 0.02 for cognitive functions, 0.06–0.65 for physical activity, and − 0.87 – − 0.61 for symptoms of attention-deficit hyperactivity disorder (ADHD). The present findings showed that, as a tool, board games can be expected to improve the understanding of knowledge, enhance interpersonal interactions among participants, and increase the motivation of participants. However, because the number of published studies in this area remains limited, the possibility of using board games as treatment for clinical symptoms requires further discussion.

## Background

A board game is a generic term for a game played by placing, moving or removing pieces on a board and that utilizes a game format in which pieces are moved in particular ways on a board marked with a pattern. Examples of board games include chess, Go, and Shogi. Research involving chess, which is played by two players on a board with 64 black and white squares and 16 pieces for each player [1], has contributed to the theoretical development of cognitive psychology [2]. For example, Burgoyne et al. [3] conducted a meta-analysis and demonstrated that chess skills are significantly and positively correlated with four broad cognitive abilities: fluid reasoning, comprehension-knowledge, short-term memory, and processing speed. Similarly, a meta-analysis by Sala and Gobet [4] found that chess instruction moderately improves the cognitive skills of children.

In contrast, Go is ancient board game that consists of simple elements (a line and circle, black and white colors, and stone and wood materials) combined with simple rules that generate subtleties that have enthralled players for millennia [5]. Go is a famous board game in Asian countries and has been used as a tool for increasing or maintaining brain activity for more than 5000 years [6]. It is currently gaining popularity in the United States and Europe [6], and Kim et al. [7] has suggested that playing Go might be effective for children with attention-deficit hyperactivity disorder (ADHD) due to its activation of hypo-aroused prefrontal cortical function and the enhancement of executive function. Lin et al. [8] conducted an intervention study using GO in patients with Alzheimer’s disease and showed that playing Go can also improve the clinical symptoms associated with depression, anxiety, and Alzheimer’s Disease. Similar to chess and Go, Shogi is a board game for two players that is also referred to as Japanese chess. Wan et al. [9] conducted an experiment with undergraduate students and found that Shogi training is related to activation in the head of the caudate nucleus. Taken together, the abovementioned findings suggest that chess, Go, and Shogi are effective ways to achieve various outcomes.

There are many board games other than chess, Go, and Shogi. For example, educational board games, such as Kalèdo, have been used to improve nutrition knowledge and promote a healthy lifestyle for children [10]. Zeedyk et al. [11] investigated the effectiveness of a board game for increasing knowledge about road safety and danger and found that the interventions were significantly effective in increasing children’s knowledge. Although the impacts of various board games have been previously examined, their effects have yet to be comprehensively reviewed. As a result, the functions and effects of board games as a whole remain unclear. Thus, the present review systematically examined the effectiveness of board games and programs that use board games.

## Methods

For the present review, a literature search based on the Preferred Reporting Items for Systematic Reviews and Meta-Analyses [12] using the PsycINFO and PubMed databases was conducted to collect findings on the effectiveness of board games and programs using board games. The keywords for the literature search were “board game” AND “trial,” and the date selected was September 13th, 2018. The search identified nine studies from PsycINFO and 32 studies from PubMed. The first author of this review performed a manual search that identified six additional studies, and 24 additional studies were extracted from Sala & Gobet [4], which conducted a meta-analysis about the benefits of chess. Duplicate studies were deleted and, ultimately, a list of references consisting of 66 articles was prepared.

**Fig. 1:**
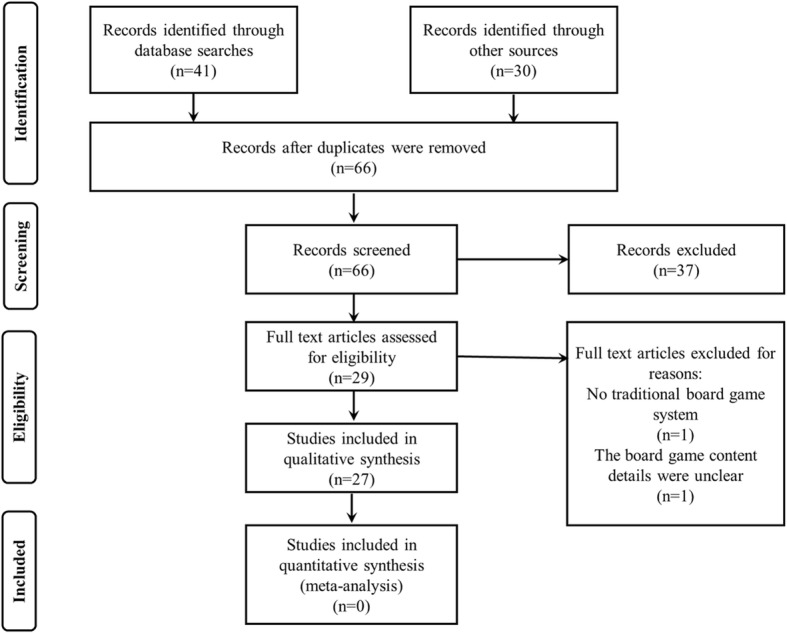
PRISMA flow chart of the study selection process

The inclusion criteria for the present study were as follows: (a) studied the effects of board games and programs using board games on psychological and educational outcomes, (b) included pre–post comparative tests, (c) used an interventional or experimental rather than a review approach, (d) had full text availability, (e) was written in English, and (f) was peer reviewed. A screening to remove articles that were judged not to satisfy any of the criteria from (a) to (f) was conducted, and 29 articles were extracted. Additionally, one study was excluded because it did not use a traditional board game (it used a Wii Fit balance board), and one study was excluded because the content details of the board game were unclear. Ultimately, 27 articles were selected for the present study; the literature search process is presented in Fig. 1.

Furthermore, in the studies where the means and standard deviations of the intervention group are described, Cohen’s d was calculated to assess effect sizes between pre- and post-tests or between pre-tests and follow-up tests with the following formula based on Cohen [13].

$$ d=\frac{M_2\hbox{-} {M}_1}{SD_{pooled}} $$

$$ {SD}_{pooled}=\sqrt{\left(\raisebox{1ex}{$\left({n}_2\hbox{-} 1\right){SD}_2^2+\left({n}_1\hbox{-} 1\right){SD}_1^2$}\!\left/ \!\raisebox{-1ex}{${n}_2+{n}_1\hbox{-} 2$}\right.\right)} $$

Note: *M*_*1*_ and *M*_*2*_ are the mean of the intervention group at the pre-test session and the post-test session or follow-up test session, respectively. *SDpooled* is the pooled standard deviation (*SD*_*1*_ is the standard deviation of the intervention group at the pre-test session and *SD*_*2*_ is the standard deviation at the post-test session or follow-up test session). *n*_*1*_ is the number of samples at the pre-test session. *n*_*2*_ is the number of samples at the post-test session or follow-up test session.

In the studies where the means and standard deviations are described in the intervention group and the other groups, Cohen’s d was also calculated to assess effect sizes compared to the other groups (control groups) with the following formula based on Sala et al. [14].

$$ d=\frac{M_{gi}\hbox{-} {M}_{gc}}{SD_{pooled\hbox{-} pre}} $$

$$ {SD}_{pooled\hbox{-} pre}=\sqrt{\left(\raisebox{1ex}{$\left({n}_i\hbox{-} 1\right){SD_{pre.i}}^2+\left({n}_c\hbox{-} 1\right){SD_{pre.c}}^2$}\!\left/ \!\raisebox{-1ex}{${n}_i+{n}_c\hbox{-} 2$}\right.\right)} $$

Note: *Mgi* and *Mgc* are the mean gain of the intervention group and the control group (other group) at the post-test session or at the follow-up test session, respectively, and *SDpooled-pre* is the pooled standard deviation of the two pre-test standard deviations. *SDpre.i* is the standard deviation of the intervention group at the pre-test session, and *SDpre.c* is the standard deviation of the control group at the pre-test session. *ni* is the number of samples in the intervention group who received the pre-test session and post-test session or the pre-test session and follow-up test session. *nc* is the number of samples in the control group who received the pre-test session and post-test session or the pre- test session and follow-up test session.

According to Cohen [13], Cohen’s d of approximately 0.20 is small, 0.50 medium, and 0.80 large.

## Results and discussion

### The effect of interventions with board games

In the present review, the selected studies were divided into the following three categories regarding the effects of board games and programs that use board games: educational knowledge (11 articles), cognitive functions (11 articles), and other conditions (five articles).

An overview of the findings about the effects of board games and programs that use board games related to educational knowledge is shown in Table 1 [10, 11, 15–23]. Board games in this category were used for the purpose of improving educational knowledge, and the effect sizes (Cohen’s d) between pre- and post-tests or between pre-tests and follow-up tests ranged from 0.12 to 1.81 and between the mean gain of the main intervention group and the other groups ranged from 0.81 to 0.93 and − 1.84 to − 1.65.

**Table 1:** Overview of the studies reporting the effectiveness of board games in educational knowledge

Authors	Design	Content of board game	Participants	Intervention	Impact	Effect size of board game between pre test and post test or follow-up test (Cohen’s d)	Effect size of board game between the mean gain of the main experimental group and the other groups (Cohen’s d)
Khazaal et al. (2013) [15]	RCT	The Pick-Klop game: it includes more than 300 cards with questions, each with three response options. The questions are about (1) smoking and tobacco history, (2) tobacco components and their biological effects, (3) reinforcement mechanisms involved in smoking addiction, (4) cognitive and behavioral mechanisms involved in the maintenance of smoking, (5) smoking cigarettes as a coping strategy, (6) costs of tobacco addiction and the benefits of quitting smoking, (7) stages of change, (8) cognitive and behavioral mechanisms involved in behavioral change, and (9) medications and treatments that help during smoking cessation. Players draw a card in one of the following categories: question, surprise, or temptation. If they answer the question cards correctly, players may gain points. Surprise cards add amusement, allowing players to obtain a gift or secret cards that allow them to help or block another player during play at the moment of their choice. The number of temptation cards which illustrate lapse and relapse processes, as well as relapse prevention strategies, increases at the end of the game board.	Participants were current daily smokers who were adults (18–65 years old). The Pick-Klop group: *n* = 120 (mean age: 33.7 ± 13.4), 2 weeks: completers *n* = 97, 3 month: completers *n* = 94. The psychoeducation group: *n* = 60 (mean age: 28.7 ± 10.8), 2 weeks: completers *n* = 43, 3 months: completers *n* = 38. The waiting list: *n* = 60 (mean age: 30.0 ± 10.0), 2 weeks: completers *n* = 47, 3 months: completers *n* = 41.	The Pick-Klop group: two sessions (1.5 h each) of the Pick-Klop game. The psychoeducation group: two sessions (1.5 h each) of psychoeducation about smoking and smoking cessation.	Scores on attitudes towards nicotine replacement therapy, attitudes towards smoking, and smoking self-efficacy improved for participants allocated to the Pick-Klop group and the psychoeducation group with respect to the waiting list.	d = 0.71 (between pre and post test) on Attitudes Towards Nicotine Replacement Therapy Scale (ANRT)-Perceived Advantage. d = 0.59 (between pre and follow-up) on ANRT-Perceived Advantage. d = 0.14 (between pre and post test) on ANRT-Drawback. d = 0.23 (between pre and follow-up) on ANRT-Drawback. d = − 0.46 (between pre and post test) on ANRT-"Don’t know” Answers. d = − 0.60 (between pre and follow-up) on ANRT-"Don’t know” Answers. d = 0.21 (between pre and post test) on Attitudes Towards Smoking Scale (ATS)- Adverse Effects of Smoking. d = 0.12 (between pre and follow-up) on ATS - Adverse Effects of Smoking. d = − 0.27 (between pre and post test) on ATS-Psychoactive Benefits of Smoking. d = − 0.26 (between pre and follow-up) on ATS-Psychoactive Benefits of Smoking. d = − 0.30 (between pre and post test) on ATS-Pleasure of Smoking. d = − 0.44 (between pre and follow-up) on ATS-Pleasure of Smoking. d = 0.15 (between pre and post test) on Smoking Self-Efficacy Questionnaire. d = 0.51 (between pre and follow-up) on Smoking Self-Efficacy Questionnaire. d = − 0.09 (between pre and post test) on Cigarettes Smoked Per Day. d = − 0.67 (between pre and follow-up) on Cigarettes Smoked Per Day.	Unable to calculate
Wanyama et al. (2012) [16]	RCT	The “make a positive start today!” board game: it is an educational board game on the uptake of knowledge about HIV and sexually transmitted infections. It is intended to increase people living with HIV’s participation and attention, to generate knowledge on HIV, sexually transmitted infections and antiretroviral treatment, and to enhance prevention behavior.	Participants were 180 patients. The intervention group: *n* = 90 (mean age: 60). The standard of care group: *n* = 90 (mean age: 55).	The intervention group played the “make a positive start today!” board game. The standard of care group participated a standardized health talk.	The intervention group which played the “make a positive start today!” board game has higher uptake of knowledge of HIV and sexually transmitted infections than the standard of care group.	Unable to calculate	Unable to calculate
Nieh & Wu (2018) [17]	cRCT	The Galaxy Rescuers game: it is designed for 2 to 6 players. The storyline of the game is about 6 characters attending the training school to become the rescuers of the Galaxy. The game includes 3 stages. At the first stage, the players earn points by answering questions about bullying. At the second stage, the players complete a mix and match game by matching characters, such as a bully, a victim, a reinforcer, or a defender and their roles in a bullying incident. The third stage is a collaborative game in which the players need to work together to accomplish their training tasks.	Participants were 328 students (11–12 years old). The game-only group: *n* = 116. The game-with-debriefing group: *n* = 125. The control group: *n* = 87.	The game-only group played the Galaxy Rescuers game. The game-with-debriefing group played the Galaxy Rescuers game and conducted reflection and discussion. The control group conducted regular bulling curriculum using conventional teaching methods, such as antibullying videos and worksheet assignments.	The Galaxy Rescuers game significantly increased players’ knowledge about bullying. The game-with-debriefing group showed a larger increase in bullying knowledge than the game-only group. The game-with-debriefing group also showed a change in bullying attitude and empathy.	Unable to calculate	Unable to calculate
Martins et al. (2018) [18]	cRCT	The board game educational intervention “Trilha Família Amamenta” (Breastfeeding Family’s Trail).	Participants were 171 children in the third grade of elementary school. Analyzed participants were 99. The intervention group: *n* = 51, post-assessment after 30th: *n* = 48. The control group: *n* = 56, post-assessment after 30th: *n* = 51.	The intervention group: children participated in the educational intervention with the board game. The control group: children did not participate in the educational intervention with the board game.	Scores for knowledge on breastfeeding were higher in the intervention group, on the 7th and 30th, than the control group. Within the intervention group, there was a significant increase of the means of scores for knowledge on breastfeeding in the posttest for the 30th day.	d = 1.50 (between pre and post test at the 7th day) on Breastfeeding Knowledge. d = 1.81 (between pre and post test at the 30th day) on Breastfeeding Knowledge.	d = 0.81 (between the mean gain of the intervention group and the control group at the 7th day) on Breastfeeding Knowledge. d = 0.93 (between the mean gain of the intervention group and the control group at the 30th day) on Breastfeeding Knowledge.
Viggiano et al. (2018) [19]	cRCT	The board game “Kaledo”: it is a new educational board game to improve nutrition knowledge and to promote a healthy lifestyle. The game is designed to be attractive for people of every age from kids to adults. A typical game session requires two to four players and lasts about 15–30 min.	Participants were 1313 children. The treatment group: *n* = 837, the first post-assessment at 8 months: *n* = 651, the second post-assessment at 18 months: *n* = 254. The control group: *n* = 476, the first post-assessment at 8 months: *n* = 356, the second post-assessment at 18 months: *n* = 202.	The treatment group: the children participated in one session (15–30 min) with the board game Kaledo, every week for 20 weeks. The control group: the children did not play with Kaledo.	The treated group significantly increased the consumption of healthy food, and decreased junk food consumption compared to the control group. The treated group significantly increased in frequency and duration of physical activity compared to the control group. The BMI z-score in the treated group significantly decreased compared to that in the control group.	Unable to calculate	Unable to calculate
Karbownik et al. (2016) [20]	RCT	The board game “AntimicroGAME” was designed to integrate bacteriology and mechanisms of action of antimicrobial drugs. The factual content of the “AntimicroGAME” was based around the existing basic medical pharmacology syllabus for the undergraduate course in medicine and further revised by an independent senior specialist in medical pharmacology.	Participants were 124 students. The board game group: *n* = 63 (mean age: 23.2 ± 1.1). The control group (lecture-based seminar): *n* = 61 (mean age: 23.6 ± 1.7).	The board game group: participants played board game “AntimicroGAME”. The control group: participants received lecture-based seminar.	The board game group significantly increases knowledge retention at post-test in final examination. Knowledge retention of board game group was higher than lecture-based seminar group.	Unable to calculate	Unable to calculate
Viggiano et al. (2015) [21]	cRCT	The board game “Kaledo”	Participants were 3110 (9–19 years old). The treatment group: *n* = 1663 (mean age: 13.3), the follow-up after 6 months: *n* = 1076, the follow-up after 18 months: *n* = 624. The control group: *n* = 1447 (mean age: 13.0), the follow-up after 6 months: *n* = 1080, the follow-up after 18 months: *n* = 421.	The treatment group: the treatment group received Kaledo each week over 20 consecutive weeks. The control group: the control group did not receive any intervention.	At the first post-assessment after 6 months, the treatment group obtained significantly higher scores than the control group on the adolescent food habits checklist (the examination of healthy eating behaviors in adolescents), nutrition knowledge, healthy and unhealthy diet and food, physical activity and lifestyle, food habits. The treated group had significantly lower BMI z-score with respect to the controls at the first post-assessment after 6 months, and second post-assessment after 18 months.	Unable to calculate	Unable to calculate
Charlier & Fraine (2013) [22]	cRCT	The educational board game in first aid: the game board is a landscape of a developing country built by the players as the game progress. The goal of the game is to build the most first aid posts and hospitals by collecting question cards (representing building material).	Participants were 120 children in general secondary. The board game group: *n* = 62. The lecture group: *n* = 58.	The board game group: participants played the board game. The lecture group: participants received a lecture about first aid with video, pictures, and demonstrations.	The board game group and the lecture group showed significantly increase in first aid knowledge. The lecture group was significantly more effective in increasing knowledge for first aid, as compared to the board game group.	d = 1.40 (between pre and post test) on Knowledge of First Aid. d = 0.78 (between pre and retention test) on Knowledge of First Aid.	d = − 1.84 (between the mean gain of the intervention group and the lecture group) on Knowledge of First Aid. d = − 1.65 (between the mean gain of the intervention group and the lecture group) on Knowledge of First Aid.
Amaro et al. (2006) [10]	cRCT	The board game “Kaledo”	Participants were 291(11–14 years old). The treatment group: *n* = 188, the complete post assessment: *n* = 153 (mean age: 12.3 ± 0.8). The control group: *n* = 103, the complete post assessment: *n* = 88 (mean age: 12.5 ± 0.7).	Treatment group: the treatment group participated Kalèdo every week in one play session (15–30 min), during 24 weeks. Control group: control group did not receive any intervention.	The treatment group showed a significant increase in nutrition knowledge and in weekly vegetable intake with respect to the control group.	Unable to calculate	Unable to calculate
Zeedyk et al. (2001) [11]	RCT (the control group only is convenience sampling)	The road safety board game: players take the part of characters who have to get home from school safely, by rolling dice and moving the playing piece around the board. In the process of getting home, characters are required to carry out a number of errands (e.g., posting a letter at the post office, returning a book to the library), each of which involves crossing the road. The players must decide the safest way of accomplishing the road crossing. The first player to get their playing piece ‘home’, having completed all the tasks safely, is declared the winner.	Participants were 120 (4–5 years old). The playmat model group: *n* = 27. The board game group: *n* = 29. The talk using illustrated posters and flip-chart materials group: *n* = 29. The control group: *n* = 35.	The playmat model group, the board game group, and the talk using illustrated posters and flip-chart materials group: each intervention was administered for only a single session, lasting approximately 20 min. The control group: control group did not receive any intervention.	Each interventions were signieficantly effctive in increasing children’s knowledge about safe and dangerous locations at which to cross the street, and that the knowledge was retained for a period of 6 months. At post-test, score of the knowledge for the control group was siginificantly lower than scores for the intervention groups.	Unable to calculate	Unable to calculate
Bartfay & Bartfay (1994) [23]	A quasi-randomized experimental study	The lifestyles board game: it is a board game (50 by 60 cm) that consists of dice, plastic tokens, six lifestyle risk factor score cards, and 40 game question cards. The games can be played by two to six individuals and requires approximately 60 min to complete. Players attempt to collect tokens awarded by the nurse to cancel the 10 lifestyle risk factors depicted on their score cards, by providing correct answers to questions on the 40 game cards.	Participants were 23 students. The board game group: *n* = 12. The control group: *n* = 11.	The board game group: the board game group participated twice, 2 weeks apart, for a period of 60 min. The control group: the control group carried on with their regularly scheduled classroom activities.	The board game group significantly increased knowledge of anatomy and physiology, diet and nutrition, and lifestyle risk factors. The gain knowledge on post-test were found to be significant between the board game group and the control group.	Unable to calculate	Unable to calculate

An overview of the findings about the effects of board games and programs that use board games on cognitive functions is shown in Table 2 [6, 24–33]. This category included board games such as Go, Ska, and chess, and the effect sizes (Cohen’s d) between pre- and post-tests of cognitive function ranged from 0.04 to 2.60 and − 1.14 to − 0.02. The effect size of the exacerbation was calculated in only the chess group of Sala et al. [27]. The effect sizes (Cohen’s d) between the mean gain of the main intervention group and the other groups ranged from 0.06 to 2.36 and − 1.38 to − 0.22.

**Table 2:** Overview of the studies reporting the effectiveness of board games in cognitive functions

Authors	Design	Content of board game	Participants	Intervention	Impact	Effect size of board game between pre test and post test or follow-up test (Cohen’s d)	Effect size of board game between the mean gain of the main experimental group and the other groups (Cohen’s d)
Iizuka et al. (2018) [6]	RCT	The board game “Go”: it is a famous board game in Asian countries, particularly Japan, China, and Korea, and it is gaining popularity in the United States and Europe.	There are 33 participants at randomization. Analyzed participants were 17. The intervention group: *n* = 9 (mean age: 89.1 ± 4.1). The control group: *n* = 8 (mean age: 89.1 ± 6.6).	The intervention group: participants received the intevention program once a week for 1 h, for a total of 15 classes. Each 1-h session consisted of a lecture on the basic rules and techniques of the game GO (15 min), solving GO game exercises (15 min), and playing games (30 min). The control group: participants received the usual care.	The intervention group showed improved attention and working memory scores, while the control group showed declines in these scores.	d = 0.13 (between pre and post test) on Montreal Cognitive Assessment. d = 0.46 (between pre and post test) on total of Digit Span Test. d = 0.49 (between pre and post test) on Digit Span Forward Task. d = 0.16 (between pre and post test) on Digit Span Backward Task.	d = 0.41 (between the mean gain of the intervention group and the control group) on Montreal Cognitive Assessment. d = 0.85 (between the mean gain of the intervention group and the control group) on total of Digit Span Test. d = 0.55 (between the mean gain of the intervention group and the control group) on Digit Span Forward Task. d = 0.57 (between the mean gain of the intervention group and the control group) on Digit Span Backward Task.
Panphunpho et al. (2013) [24]	RCT	The board game “Ska”: it is a traditional board game in Thailand. The players move the pieces in the holes provided on the board. These holes are called ‘Jooms’. Each side of the board contains 1 to 12 Jooms.	Participants were 40. The Ska group: *n* = 20 (mean age: 64.20 ± 3.22). The control group: *n* = 20 (mean age: 65.15 ± 3.19).	In the Ska group and the control group, the duration of the practice was 50 min per day, three sessions per week for the continuous duration of 16 weeks. The Ska group: the participants received Ska program. The control group: the activities of the control group included 1) Self-introduction, 2) Background telling, 3) Changes in older age, 4) Our body, 5) Food pyramid, 6) Watching television, 7) Listening to the radio, 8) Watering trees, 9) Parties, 10) Cleaning, 11) Listening to dhamma talks, 12) Diseases in the elderly, and 13) Your own health.	The Ska group showed significant better scores of cognitive functions in memory, attention, executive function compared to the control group.	d = 2.07 (between pre and post test) on Verbal Pair Association I. d = 2.60 (between pre and post test) on Verbal Pair Association II. d = 1.54 (between pre and post test) on Visual Reproduction I. d = 1.82 (between pre and post test) on Visual Reproduction II. d = − 1.02 (between pre and post test) on Trail Making Test part A. d = − 1.14 (between pre and post test) on Wisconsin Card Sorting Test. d = 0.09 (between pre and post test) on Acetylcholinesterase Activity.	d = 2.36 (between the mean gain of the intervention group and the control group) on Verbal Pair Association I. d = 2.32 (between the mean gain of the intervention group and the control group) on Verbal Pair Association II. d = 2.24 (between the mean gain of the intervention group and the control group) on Visual Reproduction I. d = 2.00 (between the mean gain of the intervention group and the control group) on Visual Reproduction II. d = − 1.20 (between the mean gain of the intervention group and the control group) on Trail Making Test part A. d = − 1.38 (between the mean gain of the intervention group and the control group) on Wisconsin Card Sorting Test. d = 0.06 (between the mean gain of the intervention group and the control group) on Acetylcholinesterase Activity.
Demily et al. (2009) [25]	RCT	The chess game	Participants were 26 with schizophrenia. The chess group: *n* = 13 (mean age: 34.7 years old). The treatment as usual group: *n* = 13 (mean age: 38.9 years old).	The chess group: the chess group practiced chess 10 times (twice per week; 60 min per session).	The chess group significantly made more perseverative errors on Wisconsin Sorting Card Test than the treatment as usual group in the pre-test assessment. But, this difference was no longer present in the second assessment. On the Stroop Test, the number of read items of chess group was significantly increased in the second assessment for the Stroop A (Colour) and C (Interference).	Unable to calculate	Unable to calculate
Sala et al. (2015) [26]	A quasi-experimental longitudinal study: two group pre-post comparative test	The chess game	Participants were 560 students in the third, fourth, and fifth grades. The intervention group: *n* = 309 (169 males and 140 females). The control group: *n* = 251 (116 males and 135 females).	The intervention group: the intervention group received a mandatory chess course (chess course and online training). The chess courses lasted between 10 and 15 h (1 or 2 h per week). The control group: the control group performed only the normal school activities without any chess-related activity.	The intervention group significantly improved mathematical problem-solving scores compared with the control group.	d = 0.34 (between pre and post test) on mathematical problem-solving scores.	d = 0.33 (between the mean gain of the intervention group and the control group) on mathematical problem-solving scores.
Sala & Gobet. (2017) [27]	A quasi-experimental longitudinal study: three group pre-post comparative test	The chess game, the checkers game and the Go game	Experiment 1 Participants were 233 students in three classes of third and fourth grades from eight Italian schools (mean age 8.50: ±0.67 years). The chess group: *n* = 53 The checker group: *n* = 82 The regular school activites group: *n* = 98 Experiment 2 Participants were 52 students in three classes of fourth grades primary school (mean age: 9.32 ± 0.32 years). The three classes were randomly assigned to three groups (a chess group, a Go group, a control group).	Experiment 1 The chess group: the participants attended 25 h of chess lessons. The checkers group: the participants attended 25 h of checkers lessons. The regular school activities group: the participants attended regular school activities only. Experiment 2 The chess group: the participants attended 15 h of chess lessons during school hours, along with regular school activities. The Go group: the participants attended 15 h of Go lessons during school hours, along with regular school activities. The regular school activities group: the participants attended regular school activities only.	Experiment 1 The results showed no siginificant differences between the three groups in mathmatical ability or metacognitive ability. Experiment 2 The chess group marginally outperformed the Go group in mathematical ability. No significant difference was found between the control and the chess group in mathematical ability. No significant differences were found between the three groups with regard to metacognition.	Experiment 1 d = 0.04 (between pre and post test) on mathematical problem-solving scores in the chess group. d = 0.30 (between pre and post test) on mathematical problem-solving scores in the checker group. d = 0.36 (between pre and post test) on mathematical problem-solving scores in the regular school activites. d = − 0.14 (between pre and post test) on matacognitive ability scores in the chess group. d = 0.07 (between pre and post test) on matacognitive ability scores in the checker group. d = 0.09 (between pre and post test) on matacognitive ability scores in the regular school activites. Experiment 2 Unable to calculate	Experiment 1 d = −0.23 (between the mean gain of the chess group and the checkers group) on mathematical problem-solving scores. d = − 0.32 (between the mean gain of the chess group and the regular school activities group) on mathematical problem-solving scores. d = − 0.22 (between the mean gain of the chess group and the checkers group) on matacognitive ability scores. d = − 0.22 (between the mean gain of the chess group and the regular school activities group) on matacognitive ability scores. Experiment 2 Unable to calculate
Aciego et al. (2012) [28]	A quasi-experimental longitudinal study: two group pre-post comparative test	The chess game	Participants were 230 students. The extracurricular activity of chess group: *n* = 170 The extracurricular activities of soccer or basketball group: *n* = 60	The extracurricular activity of chess group: the participants were conducted chess as extracurricular. The extracurricular activities of soccer or basketball group: the participants were conducted soccer or basketball as extracurricular.	The extracurricular activity of chess group significantly improved cognitive abilities (similarities, digit span object assembly mazes) and coping (identifies the problem, thinks of alternatives, assesses the alternatives, confident performance) compared to the extracurricular activities of soccer or basketball group.	d = 0.38 (between pre and post test) on similarities in Wechsler Intelligence Scale for children (WISC-R). d = 0.55 (between pre and post test) on digit span in WISC-R. d = 0.41 (between pre and post test) on object assembly in WISC-R. d = 0.38 (between pre and post test) on mazes in WISC-R. d = 0.82 (between pre and post test) on identifies the problem in coping. d = 0.71 (between pre and post test) on thinks of alternatives in coping. d = 0.77 (between pre and post test) on assesses the alternatives in coping. d = 0.65 (between pre and post test) on confident performance in coping.	d = 0.26 (between the mean gain of the extracurricular activity of chess group and the extracurricular activities of soccer or basketball group) on similarities in Wechsler Intelligence Scale for children (WISC-R). d = 0.43 (between the mean gain of the extracurricular activity of chess group and the extracurricular activities of soccer or basketball group) on digit span in WISC-R. d = 0.30 (between the mean gain of the extracurricular activity of chess group and the extracurricular activities of soccer or basketball group) on object assembly in WISC-R. d = 0.25 (between the mean gain of the extracurricular activity of chess group and the extracurricular activities of soccer or basketball group) on mazes in WISC-R. d = 0.52 (between the mean gain of the extracurricular activity of chess group and the extracurricular activities of soccer or basketball group) on identifies the problem in coping. d = 0.48 (between the mean gain of the extracurricular activity of chess group and the extracurricular activities of soccer or basketball group) on thinks of alternatives in coping. d = 0.60 (between the mean gain of the extracurricular activity of chess group and the extracurricular activities of soccer or basketball group) on assesses the alternatives in coping. d = 0.33 (between the mean gain of the extracurricular activity of chess group and the extracurricular activities of soccer or basketball group) on confident performance in coping.
Aydin (2015) [29]	A quasi-experimental longitudinal study: two group pre-post comparative test	The chess game	Participants were 26 students. The chess group: *n* = 14 (9 males and 5 females) The control group: *n* = 12 (8 males and 4 females)	The chess group: the participants were trained for chess over a 12 week-period (1 day a week, 4 h) The control group: the participants were not trained for chess.	The chess group significantly improved math scores.	d = 1.72 (between pre and post test) on math scores.	d = 1.73 (between the mean gain of the chess group and the control group) on math scores.
Barrett & Fish (2011) [30]	A quasi-experimental longitudinal study: two group pre-post comparative test	The chess game	Participants were 31 students. The treatment group: *n* = 15 (5 males and 10 females) The comparison group: *n* = 16 (6 males and 10 females)	The chess group: the students received chess instruction (1 day a week for 30 weeks) instead of the standard math curriculum. The comparison group: the students received instruction in the standard math curriculum that was individualized to meet the goals and objectives of each student’s individualized education program.	The chess group significantly improved “end of year course grades”, “number, operations and quantitative reasoning” and “probability and statistics” compared with the comparison group.	d = 0.21 (between pre and post test) on end of year course grades. d = − 0.02 (between pre and post test) on number, operations and quantitative reasoning. d = 0.13 (between pre and post test) on probability and statistics.	d = 0.84 (between the mean gain of the chess group and the compairson group) on end of year course grades. d = 0.76 (between the mean gain of the chess group and the compairson group) on number, operations and quantitative reasoning. d = 0.48 (between the mean gain of the chess group and the compairson group) on probability and statistics.
Gliga & Flesner (2014) [31]	RCT	The chess game	Participants were 38 students. The chess group: *n* = 20 (mean age: 9.85 ± 0.67, 10 males and 10 females) The control group: *n* = 18 (mean age: 9.71 ± 0.77, 10 males and 8 females)	The chess group: the participants received one training session of chess per week for 10 weeks. The control group: the participants received in a fun math program.	The chess group significantly increased school performance with respect to the control group.	Unable to calculate	Unable to calculate
Hong & Bart (2007) [32]	RCT	The chess game	Participants were 38 students. The chess group: *n* = 18 (mean age: 9.71, 12 males and 6 females) The control group: *n* = 20 (mean age: 9.74, 15 males and 5 females)	The chess group: the participants received a 90 minute chess lesson once per week over a three-month period. The control group: the participants regularly attended school activities after class.	The chess group performance on the nonverbal abilities was not different from the control group performance.	d = 0.29 (between pre and post test) on Test of Nonverbal Intelligence-Third Edition.	d = − 0.39 (between the mean gain of the chess group and the control group) on Test of Nonverbal Intelligence-Third Edition.
Scholz et al., (2008) [33]	A quasi-experimental longitudinal study: two group pre-post comparative test	The chess game	Participants were 53 students. The chess group: *n* = 31 The control group: *n* = 22	The chess group: the participants received 1 hour of chess lesson instead of 1 hour of regular mathmatics lessons per week for the duration of one school-year. The control group: the participants received the planned five regular lessons of mathematics per week.	Calculation abilities for simple addition tasks and counting improved significantly more in the chess group than in the control group.	Unable to calculate	Unable to calculate

An overview of the findings about the effects of board games and programs that use board games on other conditions is shown in Table 3 [7, 8, 34–36]. This category addressed the impacts of board games on physical activity, anxiety, ADHD symptoms, and the severity of Alzheimer’s Disease. The effect sizes (Cohen’s d) between pre- and post-tests or between pre-tests and follow-up tests ranged from 0.06 to 0.65 for physical activity and from − 0.87 to − 0.61 for ADHD symptoms.

**Table 3:** Overview of the studies reporting the effectiveness of board games in the other conditions

Authors	Design	Content of board game	Participants	Intervention	Impact	Effect size of board game between pre test and post test or follow-up test (Cohen’s d)	Effect size of board game between the mean gain of the main experimental group and the other groups (Cohen’s d)
Mouton et al. (2017) [34]	A quasi-experimental longitudinal study: two group pre-post comparative test	The giant exercising board game: it required participants to perform strength, flexibility, balance and endurance activities. The tarpaulin surface was printed with 24 numbered squares of 50 × 50 cm and surrounded by a walking lane. Each square was colored according to the component of physical fitness that was to be performed (ie, 6 squares/component): strength, flexibility, balance, and endurance. The rules were simple and made available to the participants in a folder adjacent to the mat. Taking turns, participants turned the wheel and had reach the next square with the color targeted by the arrow. After completing the requested exercises, participants were expected to do systematically two laps on the walking lane. Participants made their way through the squares until the finish line after the 24th square. The playing time of a session ranged between 30 and 60 min and the game requires a minimum of 2 participants.	Participants were 21. The intervention group: *n* = 10 (mean age: 82.5 ± 6.3), the post-intervention: *n* = 9, the follow-up after 3 months: *n* = 8. The control group: *n* = 11 (mean age: 89.9 ± 3.1), the post-intervention: *n* = 10, the follow-up after 3 months: *n* = 9.	The intervention group: 4 supervised exercising sessions were planned on the board game during the first week and then 3, 2, and 1 sessions were planned during the second, third, and fourth week of the intervention. The control group: participants in the control group were requested neither to change their lifestyle during the study nor to get involved in any new type of physical activity.	The intervention group significantly increases steps per day (number), energy expenditure per day and quality of life and improves body balance and gait abnormalities, the strength of ankle extensors and flexors.	d = 0.06 (between pre and post test) on Steps Per Day (number). d = 0.50 (between pre and follow-up) on Steps Per Day (number). d = 0.36 (between pre and post test) on Tinetti Test. d = 0.65 (between pre and follow-up) on Tinetti Test.	Unable to calculate
Fernandes et al. (2014) [35]	RCT	The educational board-game: in this study, the educational materials are composed of seven parts, illustrating the hospital stages: (i) Hospital admission; (ii) Healthcare professionals and hospital rules; (iii) Medical instruments; (iv) Medical procedures; (v) Anesthesia and Surgery room; (vi) Recovery room; and (vii) Aftercare and Going home. Each part is composed of clear explanations about specific topics and intervention stages (e.g. information about healthcare professionals, medical instruments, clinical procedures and induction of anesthesia), as well as explanations of specific hospital and medical rules (e.g. reasons why they should not eat or drink before surgery, the changing of clothes and parental separation during surgery). These educational information was provided though a board game.	Participants were 125 children (mean age: 10.09 ± 1.43). The experimental group: *n* = 45 (mean age: 10.29 ± 1.25), the educational booklet (n = 15), the educational video (*n* = 15), the educational board-game (*n* = 15). The comparison group: *n* = 45 (mean age: 9.84 ± 1.48), the entertainment booklet (*n* = 15), the entertainment video (*n* = 15), the entertainment board-game (*n* = 15). The control group: *n* = 35 (mean age: 10.14 ± 1.57).	The experimental group: participants received educational materials about surgery and hospitalization in the format of a board game, a video or a booklet. The comparison group: participants received materials in the format of a board game, a video or a booklet but the materials contained no information about surgery or hospitalization. The control group did not receive any material.	Children in the experimental group showed significant lower preoperative worries than children in both the comparison group and the control group. Children received the educational board-game showed lower preoperative worries than children received the entertainment booklet, video, or board-game group.	Unable to calculate	Unable to calculate
Blasco-Fontecilla et al. (2016) [36]	A quasi-experimental longitudinal study: one pre-post comparative test	The chess game	Participants were 44 children with Attention deficit hyperactivity disorder (ADHD) (6–17 years old).	All children had weekly 1 h sessions over a period of 11 consecutive weeks taught by a chess expert. Participants took chess training lessons in groups of up to 10 individuals.	Children with ADHD significantly decreased in the severity of ADHD (both inattention and hyperactivity-impulsivity).	d = − 0.85 (between pre and post test) on Swanson, Nolan and Pelham Scale for parents (SNAP-IV)-total. d = − 0.87 (between pre and post test) on SNAP-IV-Inattention. d = − 0.61 (between pre and post test) on SNAP-IV-Hyperactivity-Impulsivity. d = − 0.86 (between pre and post test) on Abbreviated Conner’s Rating Scales for parents.	Unable to calculate
Kim et al. (2014) [7]	A quasi-experimental longitudinal study: two group pre-post comparative test	The Go game	Participants were 34 children. The ADHD group: 17 drug-naïve children with ADHD (mean age: 10.1 ± 1.5). The control group: 17 age- and sex-matched comparison subjects with-out ADHD (mean age: 10.2 ± 1.6).	During the 16 weeks, both ADHD children without medication and children of control group were asked to learn and play Go for 2 hours/day with an instructor of the game of Go. Participants played Go under the instructor’s education for 2 h a day during weekday, Monday to Friday. Go training with the same protocol had been provided.	There were significant improvement of severity of ADHD symptoms in children with ADHD. Children with ADHD ameliorated cognitive flexibility and cognitive persistence. The change of theta/beta right of prefrontal cortex during 16 weeks was greater in children with ADHD than children of the control group.	d = 0.57 (between pre and post test) on total of Digit Span Test. d = 0.80 (between pre and post test) on Digit Span Forward Task. d = 1.33 (between pre and post test) on Digit Span Backward Task. d = 1.28 (between pre and post test) on Children’s Trails Test (CCTT)-1. d = − 0.23 (between pre and post test) on CCTT-2. Unable to calculate about the severity of ADHD	d = 1.18 (between the mean gain of the intervention group and the control group at the post test) on total of Digit Span Test. d = 0.88 (between the mean gain of the intervention group and the control group at the post test) on Digit Span Forward Task. d = 1.28 (between the mean gain of the intervention group and the control group at the post test) on Digit Span Backward Task. d = 0.32 (between the mean gain of the intervention group and the control group at the post test) on Children’s Trails Test (CCTT)-1. d = − 0.29 (between the mean gain of the intervention group and the control group at the post test) on CCTT-2. Unable to calculate about the severity of ADHD
Lin et al. (2015) [8]	RCT	The Go game	Participants were 147 patients. The short-time group: *n* = 49. The long-time group: *n* = 49. The control group: *n* = 49.	The short-time group: the short-time group play Go 1 h daily for 6 months. The long-time group: the long-time group play Go 2 h daily for 6 months. The control group: the control group play doesn’t play Go.	Playing Go improved depression and anxiety and ameliorated Alzheimer Disease manifestations by up-regulating brain derived neurotrophic factor levels.	Unable to calculate	Unable to calculate

### Board games and educational knowledge

Eleven studies that used board games to increase educational knowledge were selected for this review. The present findings showed that board games influence educational knowledge and concomitant outcomes, with the effect sizes for educational knowledge ranging from very small to large.

Board games can be used as a tool to encourage learning. In previous studies, specialized board games aimed at improving knowledge in the field of education were targeted and subsequently developed and investigated. For example, Wanyama et al. [16] conducted a study of the Make a Positive Start Today game, which is a board game aimed at improving knowledge about human immunodeficiency virus (HIV) and sexually transmitted infections (STIs). Similarly, Kalèdo is an educational board game used to increase nutrition knowledge [10, 19, 21]. It has been shown that these board games contribute to increasing knowledge related to each particular field.

Board games are also efficacious for goals other than increasing knowledge. According to Charlier and De Fraine [22], board games can be an enjoyable and motivational method for learning content and enhancing group interactions, competition, and fun. Martins et al. [18] reported that board games teach educational content in a playful and enjoyable way and involve interactions with family and friends; thus, they favor knowledge acquisition by enabling exchanges of experiences and learning. Furthermore, Wanyama et al. [16] showed that, as a method of health education, board games increase the acquisition of knowledge as well as result in more positive experiences than do health talks among both participants and facilitators. Amaro et al. [10] found that class teachers noted improvements in student interest and appreciation of the board game. Taken together, these findings suggest that board games may improve the motivation of participants. Furthermore, Karbownik et al. [20] showed that a board game was warmly welcomed by students; in their opinion, it facilitated clinical thinking and peer communication. Therefore, board games may also have a positive influence on interpersonal interactions among participants.

Based on the above findings, board games can be used as a tool to encourage learning as well as to enhance motivation and interpersonal interactions. In clinical treatment, it is important to increase motivation because low motivation to cooperate with a particular intervention may lead to a patient dropping out of treatment or to interference with the therapeutic effects. Based on the above findings, the use of board games may help increase the benefit of treatment for less motivated patients.

### Board games and cognitive functions

In the present review, 11 of the assessed studies investigated the effects of board games and programs that use board games on cognitive functions. These studies used Go, chess, and Ska, which are not educational games but abstract strategy games. Studies investigating the use of Go found that older adults experiencing cognitive decline and/or living in nursing homes showed improvements in attention and working memory after regularly playing the game [6]. Studies assessing the use of Ska found that the game appeared to enhance the cognitive functioning of older adults in terms of memory, attention, and executive function [24]. Studies evaluating chess showed that training with the game improved the planning ability of patients with schizophrenia and the mathematical ability of children [25, 26]. But, Sala & Gobet [27] indicated that interventions that use chess are not significantly different from interventions that use checkers and regular school activities that address the mathematical and metacognitive ability of children.

The effect sizes for cognitive functions ranged from very small to large, but the effect size of exacerbation on metacognitive ability was shown in the chess training of Sala & Gobet [27]. The number of studies included in this category was relatively limited. Further investigations will be necessary to clarify the more detailed effects of board games on cognitive function. Articles about Shogi were not selected for this category in the present review. Because Shogi was also included with the abstract strategy games, this may influence cognitive functions. In the future, it will be necessary to use intervention studies to examine the effects of additional types of board games, including Shogi, on cognitive function.

### Board games and other conditions

The “other studies” category in the present review included five studies that examined the effects of board games on physical activity, physical and psychological outcomes, ADHD symptoms, and the severity of Alzheimer’s Disease. Mouton et al. [34] showed that a giant board game intervention for nursing home residents led to significant increases in ambulatory physical activity, daily energy output, quality of life, balance and gait, and ankle strength. The effect sizes in the present review of studies related to physical activity ranged from very small to medium. Fernandes et al. [35] reported that board games used as educational preoperative materials decreased the preoperative anxiety of children. Additionally, the use of board games contributed to improvements in the ADHD symptoms of children [7, 35]. The effect sizes for ADHD symptoms in the present review ranged from medium to large. Lin et al. [8] showed that playing Go improved the symptoms of depression and anxiety and ameliorated the manifestations of Alzheimer’s Disease. Although a study by Barzegar and Barzegar [37] was not selected for the present review because it was a case report, these authors found that playing chess prevented panic attacks and contributed to the amelioration of this condition. Taken together, these findings indicate that board games might be an effective complementary intervention for the treatment of the clinical symptoms of ADHD and Alzheimer’s Disease.

In terms of Alzheimer’s disease, board games may also play a role in the prevention of the onset of this disorder. According to an epidemiological survey in Japan [38], the prevalence rates of dementia in 1980, 1990, and 2000 were 4.4, 4.5, and 5.9, respectively, for all types of dementia and 1.9, 2.5, and 3.6, respectively for Alzheimer’s Disease. In Japan, the number of patients with Alzheimer’s disease has increased, and the prevention of this disorder is a problem that must be addressed. Because playing board games ameliorates the manifestations of Alzheimer’s disease [8], these types of games may contribute to the prevention of this disorder. However, the number of studies in the present review that investigated the effects of board games on clinical symptoms was quite small, and further research will be required.

### Possible clinical applications of board games

It is also important to note that board games can be played without the use of language. Language-based therapies may not be appropriate for people with underdeveloped linguistic functions, such as children and patients with speech disorders. However, board games may be a viable treatment option for these populations. In the present review, the subjects in 18 of the assessed studies included children, which is a group that is still developing linguistic functions and is more likely to have poor knowledge about diseases. The present review also revealed that board games and programs that use board games are effective for achieving various outcomes for children, including increasing educational knowledge, enhancing cognitive functions, and decreasing anxiety and the severity of ADHD. Furthermore, board games can be an enjoyable and motivational tool for children [22]. Based on these findings, it is possible that board games can be a useful intervention for children in particular because such games can be expected to result in the maintenance and promotion of health and the prevention of disease.

### Limitations and future directions

Several limitations of the present study must be considered. First, the number of studies assessed in the present review was rather limited. Therefore, further investigations of the effects of board games will be necessary. Second, many of the papers selected for the present review examined the effectiveness of board games by comparing pre- post intervention for a single group or by comparing with a control group without intervention. These research designs do not control for the possibility of placebo effects. Intervention studies must include an active control group to control for possible placebo effects [39], thus it will be necessary to compare the effect of board game groups and active control groups in future research. Third, in the articles selected for the present review, some studies were conducted with relatively small sample sizes. In cases in which the sample size is small, there is the possibility of increased sampling error. In order to reduce sampling error, it is necessary to do a power analysis to set an appropriate sample size in intervention studies. In addition, it is desirable that multiple assessment indicators be used to examine the effects of board games in various perspectives and to reduce measurement errors.

## Conclusions

The present systematic review showed that board games and programs that use board games have positive effects on various outcomes, including educational knowledge, cognitive functions, physical activity, anxiety, ADHD symptoms, and the severity of Alzheimer’s Disease. Additionally, board games were shown to contribute to improving these variables, enhancing the interpersonal interactions and motivation of participants, and promoting learning. Taken together, these findings suggest that board games would be an effective complementary therapy that would contribute to the improvement of many clinical symptoms.

## Data Availability

Not applicable.

## Abbreviations

ADHD: Attention-deficit hyperactivity disorder
cRCT: cluster randomized controlled trial
HIV: Human immunodeficiency virus
RCT: Randomized controlled trial
STIs: Sexually transmitted infections

## References

[1] El Daou BMN, El-Shamieh SI (2015). The effect of playing chess on the concentration of ADHD students in the 2nd cycle. Procedia Soc Behav Sci.

[2] Charness N (1992). The impact of chess research on cognitive science. Psycho Res.

[3] Burgoyne AP, Sala G, Gobet F, Macnamara BN, Campitella G, Hambrick DZ (2016). The relationship between cognitive ability and chess skill: a comprehensive meta-analysis. Intelligence.

[4] Sala G, Gobet F (2016). Do the benefits of chess instruction transfer to academic and cognitive skill?: a meta-analysis. Educ Res Rev.

[5] American Go Association. What is Go? American Go Association. Retrieved from http://www.usgo.org/what-go (January 22, 2019).

[6] Iizuka A, Suzuki H, Ogawa S, Kobayashi-Cuya KE, Kobayashi M, Fujiwara Y (2018). Pilot randomized controlled trail of the GO game intervention on cognitive function. Am J Alzheimers Dis Other Dement.

[7] Kim SH, Han DH, Lee YS, Kim BN, Cheong JH, Han SH (2014). Baduk (the game of go) improved cognitive function and brain activity in children with attention deficit hyperactivity disorder. Psychiatry Investig.

[8] Lin Q, Cao Y, Gao J (2015). The impacts of a GO-game (Chinese chess) intervention on Alzheimer disease in a northeast Chinese population. Front Aging Neurosci.

[9] Wan X, Takano D, Asamizuya T, Suzuki C, Ueno K, Cheng K, Ito T, Tanaka K (2012). Developing intuition: neural correlates of cognitive-skill learning in caudate nucleus. J Neurosci.

[10] Amaro S, Viggiano A, Di Costanzo A, Madeo I, Viggiano A, Baccari ME, Marchitelli E, Raia M, Viggiano E, Deepak S, Monda M, De Luca B (2006). Kalèdo, a new educational board-game, gives nutritional rudiments and encourages healthy eating in children: a pilot cluster randomized trial. Eur J Pediatr.

[11] Zeedyk MS, Wallace L, Carcary B, Jones K, Larter K (2001). Children and road safety: increasing knowledge does not improve behavior. Br J Educ Psychol.

[12] Moher D, Liberati A, Tetzlaff J, Altman DG (2009). Preferred reporting items for systematic reviews and meta-analyses: the PRISMA statement. PLoS Med.

[13] Cohen J (1988). Statistical power analysis for the behavioural sciences.

[14] Sala G, Aksayli ND, Tatlidil KS, Gondo Y, Gobet F. Working Memory training does not enhance older adults’ cognitive skills: a comprehensive meta-analysis. from file:///C:/Users/noras/AppData/Local/Packages/Microsoft.MicrosoftEdge_8wekyb3d8bbwe/TempState/Downloads/WMT_older_adults_posting_version%20(1).pdf (July 22, 2019).

[15] Khazaal Y, Chatton A, Prezzemolo R, Zebouni F, Edel Y, Jacquet J, Ruggeri O, Burnens E, Monney G, Protti AS, Etter JF, Khan R, Cornuz J, Zullino D (2013). Impact of a board-game approach on current smokers: a randomized controlled trial. Subst Abuse Treat Prev Policy.

[16] Wanyama JN, Castelnuovo B, Robertson G, Newell K, Sempa JB, Kambugu A, Manabe YC, Colebunders R (2012). A randomized controlled trial to evaluate the effectiveness of a board game on patients' knowledge uptake of HIV and sexually transmitted diseases at the infectious diseases institute, Kampala, Uganda. J Acquir Immune Defic Syndr.

[17] Nieh HP, Wu WC (2018). Effects of a collaborative board game on bullying intervention: a group-randomized controlled trial. J Sch Health.

[18] Martins FDP, Leal LP, Linhares FMP, Santos AHDS, Leite GO, Pontes CM (2018). Effect of the board game as educational technology on schoolchildren’s knowledge on breastfeeding. Rev Lat Am Enfermagem.

[19] Viggiano E, Viggiano A, Di Costanzo A, Viggiano A, Viggiano A, Andreozzi E, Romano V, Vicidomini C, Di Tuoro D, Gargano G, Incarnato L, Fevola C, Volta P, Tolomeo C, Scianni G, Santangelo C, Battista R, Raia M, Valentino I, Palumbo M, Messina A, Monda M, De Luca B, Amare S (2018). Healthy lifestyle promotion in primary schools through the board game Kaledo: a pilot cluster randomized trial. Eur J Pediatr.

[20] Karbownik MS, Wiktorowska-Owczarek A, Kowalczyk E, Kwarta P, Mokros Ł, Pietras T (2016). Board game versus lecture-based seminar in the teaching of pharmacology of antimicrobial drugs–a randomized controlled trial. FEMS Microbiol Lett.

[21] Viggiano A, Viggiano E, Di Costanzo A, Viggiano A, Andreozzi E, Romano V, Rianna I, Vicidomini C, Gargano G, Incarnato L, Fevola C, Volta P, Tolomeo C, Scianni G, Santangelo C, Battista R, Monda M, Viggiano A, De Luca B, Amaro S (2015). Kaledo, a board game for nutrition education of children and adolescents at school: cluster randomized controlled trial of healthy lifestyle promotion. Eur J Pediatr.

[22] Charlier N, De Fraine B (2013). Game-based learning as a vehicle to teach first aid content: a randomized experiment. J Sch Health.

[23] Bartfay WJ, Bartfay E (1994). Promoting health in schools through a board game. West J Nurs Res.

[24] Panphunpho S, Thavichachart N, Kritpet T (2013). Positive effects of Ska game practice on cognitive function among older adults. J Med Assoc Thail.

[25] Demily C, Cavézian C, Desmurget M, Berquand-Merle M, Chambon V, Franck N (2009). The game of chess enhances cognitive abilities in schizophrenia. Schizophr Res.

[26] Sala G, Gorini A, Pravettoni G (2015). Mathematical problem-solving abilities and chess: an experimental study on young pupils. SAGA Open.

[27] Sala G, Gobet F (2017). Does chess instruction improve mathematical problem-solving ability?: two experimental studies with an active control group. Learn Behav.

[28] Aciego R, Garcia L, Betancort M (2012). The benefits of chess for the intellectual and social-emotional enrichment in schoolchildren. Span J Psychol.

[29] Aydin M (2015). Examining the impact of chess instruction for the visual impairment on mathematics. Educ Res Rev.

[30] Barrett DC, Fish WW (2011). Our move: using chess to improve math achievement for students who receive special education services. Int J Special Educ.

[31] Gliga F, Flesner PI (2014). Cognitive benefits of chess training in novice children. Procedia Soc Behav Sci.

[32] Hong S, Bart WM (2007). Cognitive effects of chess instruction on students at risk for academic failure. Int J Special Educ.

[33] Scholz M, Niesch H, Steffen O, Ernst B, Markus L, Witruk E, Schwarz H (2008). Impact of chess training on mathematics performance and concentration ability of children with learning disabilities. Int J Special Educ.

[34] Mouton A, Gillet N, Mouton F, Van Kann D, Bruyère O, Cloes M, Buckinx F (2017). Effects of a giant exercising board game intervention on ambulatory physical activity among nursing home residents: a preliminary study. Clin Interv Aging.

[35] Fernandes SC, Arriaga P, Esteves F (2014). Providing preoperative information for children undergoing surgery: a randomized study testing different types of educational material to reduce children's preoperative worries. Health Educ Res.

[36] Blasco-Fontecilla H, Gonzalez-Perez M, Garcia-Lopez R, Poza-Cano B, Perez-Moreno MR, de Leon-Ma V, Otero-Perez J (2016). Efficacy of chess training for the treatment of ADHD: a prospective, open label study. Rev Psiquiatr Salud Ment.

[37] Barzegar K, Barzegar S (2017). Chess therapy: a new approach to curing panic attack. Asian J Psychiatr.

[38] Wakutani Y, Kusumi M, Wada K, Kawashima M, Ishizaki K, Mori M, Mori N, Ijiri T, Adachi Y, Ashida Y, Kuno N, Urakumi K, Takeshima T, Nakashima K (2007). Longitudinal changes in the prevalence of dementia in a Japanese rural area. Psychogeriatrics.

[39] Sala G, Aksayli ND, Tatlidil KS, Tatsumi T, Gondo Y, Gobet F (2019). Near and fear transfer in cognitive training: a second-order meta-analysis. Collabra Psychol.

